# Supplementary material for: Effects of Catha Edulis Forsk on spatial learning, memory and correlation with serum electrolytes in wild-type male white albino rats
Source: PLoS One. 2022 Feb 14;17(2):e0257932. doi: 10.1371/journal.pone.0257932 (PMC8843189; doi:10.1371/journal.pone.0257932)
Supplement: S1 Abstract — (DOCX) [file pone.0257932.s001.docx]

**Abstract**

**Background**: The burdens of psychostimulant use disorders are becoming a worldwide problem. One of the psychostimulants widely consumed in Ethiopia and East African countries is *Catha edulis* Forsk (khat). However, no studies have been conducted on the cognitive effects of khat and its correlation with serum electrolytes. The present study was aimed to evaluate the effects of khat on spatial learning and memory and their correlation with serum electrolytes.

**Materials and Methods**: Diethyl ether and chloroform (3:1v/v ratio) were solvents used to obtain the crude khat extract in this study.T80W was used to prepare the khat juice, fresh khat leave extract. The rats were received crude khat extract subchronically (KESC) (100 mg/kg, 200 mg/kg and 300 mg/kg b.w), khat juice (KHJ 2.5 mL/kg), 2% tween 80 in distilled water (T80W- v/v, vehicle) and khat extract subacutely (KESA) (300 mg/kg). For subchronic treatment, each rat was administered for twelve weeks before Morris water maze experiment has been started, while it was administered for a week for acute treatment. Spatial learning and memory were measured using the Morris water maze model and serum sodium, calcium, potassium, and chloride were evaluated using Cobas 6000.

**Results**: Spatial learning was improved with trials across the groups, while average escape latency (s) of rats received KESC 200 mg/kg (p<0.001), KESC 300 mg/kg (p<0.01) and KHJ 2.5 mL/kg (p<0.05) was significantly greater than rats that received vehicle. There was no significant difference in the latency between rats that received KESA 300mg/kg and vehicle (p>0.05). Thigmotaxis was significantly higher in rats that received all doses of khat extract (p<0.001). The time spent in the target quadrant in rats that received KESC 300 mg/kg was significantly reduced (p<0.05). Serum calcium level was inversely correlated with the escape latency (R= -0.417, p<0.05) in rats that received khat.

**Conclusions**: khat extract and juice administered subchronically, but not subacutely, impaired learning and memory and was associated with serum calcium reduction. The neuronal basis for such alteration should be investigated.

**Keywords:** khat**,** Morris water maze, escape latency, time spent in the target quadrant, serum electrolytes
