# Supplementary material for: Effects of Catha Edulis Forsk on spatial learning, memory and correlation with serum electrolytes in wild-type male white albino rats
Source: PLoS One. 2022 Feb 14;17(2):e0257932. doi: 10.1371/journal.pone.0257932 (PMC8843189; doi:10.1371/journal.pone.0257932)
Supplement: S1 File — (DOCX) [file pone.0257932.s003.docx]

Date: January 8, 2022

**Response letter for the manuscript editor**

**Dear professor** Dr Solna Carreon Santos

Editorial Department

Journal of PLOS-ONE

**Subject**: **Submission of revised paper manuscript**

We authors would like to acknowledgement the reviewers for their critical looking at the article and constructive comments for possible publication. We also appreciate the editor for your great contribution and considered our manuscript entitled “**Effects of *Catha Edulis* Forsk on Spatial Learning, Memory and Correlation with Serum Electrolytes in Wild- Type Male White Albino Rats”** (PONE-D-21-29239R1) for possible publication if the comments are well addressed and satisfied the editors and reviewers. We have carefully reviewed the comments and have revised the manuscript and abstract accordingly. Our responses are given in a point-by-point manner in the table uploaded. We tried to address all the specific comments from the editor and reviewers. We hope the revised version is now suitable for publication and look forward to hear from you in due course. If you need further information about it, we are waiting for you.

Sincerely

Abebaye Aragaw Limenie

Department of Physiology, Addis Ababa University, College of Health Sciences, Addis Ababa, Ethiopia P.O. Box 9086, email, [abebaye.aragaw@aau.edu.et](mailto:abebaye.aragaw@aau.edu.et)

On the behalf of co-authors
