# Supplementary material for: Effects of Catha Edulis Forsk on spatial learning, memory and correlation with serum electrolytes in wild-type male white albino rats
Source: PLoS One. 2022 Feb 14;17(2):e0257932. doi: 10.1371/journal.pone.0257932 (PMC8843189; doi:10.1371/journal.pone.0257932)
Supplement: Attachment — Submitted filename: Point by point responses to the reviewers.docx [file pone.0257932.s004.docx]

**Point-by-point responses**

| **Reviewer I** | | |
| --- | --- | --- |
| Comments/Questions | Response | Page /line |
| **Abstract** | | |
| English editing | It has now been edited for the English language to our level best. | The whole document |
| In the’ ***Catha Edulis* Forsk ‘** Forsk should not be italicized | Now is corrected | Title page and in the abstract section (line-2) |
| The objective need to be revised and more specific | Yes, cognitive function is more general that includes thinking, concentration, attention, learning, and memory. Now it has been made more specific as ‘**The present study was aimed to evaluate the effects of khat on spatial learning and memory and their correlation with serum electrolytes’** instead of ‘The present study was aimed to evaluate the effects of khat on **cognitive functions** and its correlation with serum electrolytes. This is also corrected in the title as the title is also need to be specific | Abstract line 4-5 |
| The number and the weight of animals can be removed rather the solvents used to get the crude extract should be mentioned. What is **khat juice**? Make clear how long ‘**subchroni**c’ and ‘**subacute’** treatment last. What electrolytes are evaluated? | The solvents used in this study were diethyl ether and chloroform (3:1v/v ratio).  The khat jcts of che juicele (5-6, hs whos ical from his sabats uice was prepared from the fresh khat leaves. The fresh khat leaves in 2% tween 80 in distilled water was crushed and juice prepared. In the chronic group, each rat received the extract for twelve weeks, while it was for one week in the subacute groups. | Solvents used mentioned in the method section of the abstract (line 1-2).  Duration of treatment in the subchronic and subacute treatment also mentioned in the method section of the abstract (line 5-6).  These are also mentioned in the method section of the manuscript (page 5), under the **‘Grouping and dosing’** subheading |
| **Suggestion**: Instead of ‘kesc’, ‘kesa’ and ‘khj’, better to be written as ‘KESC’, ‘KESA’ and ‘KHJ’ in the abstract and the whole article | Corrected according to the suggestion | In the Abstract, Method (line3-6) and result sections( line2, 4 and 6) |
| **Introduction** | | |
| How khat is popularly used as a psychostimulant? | As cited in this article (5, 7, 8, 9, 18), khat is popularly used in different countries mainly the East African countries including Ethiopia. Different groups of the population in Ethiopia including students highly consume it (reference N^O^ 18).  On the other hand, one of the determinant factors for chewing khat is its stimulatory effect. According to our previous study (<https://pubmed.ncbi.nlm.nih.gov/32273781/>), khat showed a rewarding response, indicating that it has the psychostimulant effect and another review (reference N^O^ 6) showed its Psychopharmacological consequences. | Page 2, starting from line 6-10 |
| Do the authors try to investigate the presence of cathinone in the sample? | **No**: there are many studies conducted previously showing the presence of cathinone and we have used the same plant and solvents, indicating that cathinone is also found in our extract.  On the other hand, the purpose of this study was not to investigate the presence of cathinone, cathine, or other phytochemicals in the crude extract, but was to see the effect of the crude extract from khat leaves on spatial learning and memory. Naturally, Users pick the leaves, chew them on one side of the mouth, and swallow the crude extract from the leaves, so we have tried to mimic what the users naturally use. |  |
| The role of electrolytes in the cognitive function of the nervous system should be explored in the introduction | Now is included | In the introduction section of the manuscript (Page 2-3, starting from line 17-29 |
| Clear the sentence ‘**Regardless of its adverse effect’ (8 and 9), misperceptions of its learning and memory effects are reflected among students**”. | We have tried to make this sentence clear | Page 1 ( line 5-7) |
| **MATERIALS AND METHODS** | | |
| 2.3. Preparation of khat juice need elaboration and Amount of T80 used? | Now is elaborated  The 2% in distilled water is T80 (1:50, volume-to-volume ratio | Page 4 |
| 2.4. Approval number/protocol by the ethics committee? | Yes and this is the approval number:012/15/physio |  |
| 2.5) What really means “during the experiment”? | during the experiment mean that this group of rats was received the extract during the Morris water maze experiment, not before the Morris water maze, other groups were administered with the extract both before the Morris water maze has started and during the Morris water maze experiment | Page 5 |
| Revise the sentence “The dose of the extract administered in each rat was calculated from the selected doses | Well elaborated | Page 5, the last paragraph, line 2-5 |
| **Results** | | |
| The lack of analysis of the chemical composition of the samples is an important negative point | Yes, it was important if we had analyzed the phytochemicals in the extract. But people use the crude not the separate components in the crude extract, and the main objective of this study is to evaluate the effect of the crude extract and juice on spatial learning and memory |  |
| Quality of the images seem bad in the version sent for review | We have used SPSS and Microsoft excel for figures |  |
| Many of the figures can be merged in one. | Figures are seemed like many and need to be merged, but each figure explains one variable such as fig 1: is about the escape latency, fig 2: is about swim-path-length, and so on |  |
| **Discussion** | | |
| Please avoid an extensive repeat of the results. | Corrected |  |
| - Again, the lack of chemical analysis makes the discussion a little in-depth and very descriptive. | This study aims to evaluate the effect of the crude extract and juice on spatial learning and memory, not analyze the chemicals found in the khat leaves extract, several studies have been done before on the chemical analysis. |  |
| There are several comparisons with other studies performed with khat. However, it should be clarified the samples evaluated in all these studies. Extracts? What types? Compounds? | The comparisons were made mostly with researches that have been conducted before on the crude effect of khat extract. For instance, similar to our study, Mohammed *et al* (2014) use the crude extract and the same solvent that we used to. The same is true in the study conducted by Shewamene and Engidawork (2014). |  |

| **Reviewer ii** | | |
| --- | --- | --- |
| Comments/questions | Response |  |
| The subject of the manuscript: **interesting** |  |  |
| Text: - **clear and easy to read** |  |  |
| Research design: **appropriate** |  |  |
| Results: - **Consistent and clearly presented** |  |  |
| The presented data:-**supported by the conclusions** |  |  |
| References: **variously** | Now is corrected and made consistent |  |
| At the reference list, the name of the species Catha edulis is **not written in italic** | Now is **italicized** (reference N^O^ 6,7,9,17,,21, 22,23,25,28, 43 and 48: marked in the reference section of the manuscript) |  |
